# Building the gulf of opinions on the health and biological effects of electromagnetic radiation

**DOI:** 10.3389/fpubh.2025.1589021

**Published:** 2025-07-23

**Authors:** Paul Héroux

**Affiliations:** ^1^International Commission on the Biological Effects of Electromagnetic Fields (ICBE-EMF), Electromagnetic Safety Alliance, Inc., Tempe, AZ, United States; ^2^Department of Epidemiology, Biostatistics and Occupational Health, McGill University, Montreal, QC, Canada

**Keywords:** health effects, electromagnetic radiation, ELF, RF, biophysics

## Abstract

Using events that the author was personally involved with over many years, the article attempts to explain how different views solidified over time on the health effects of electromagnetic radiation, some believing they are negligible, while others believe they are substantial.

## Introduction

The health impacts of Non-Thermal Electromagnetic Radiation, both in the ELF and RF domains, have been controversial since the early 1980s (1). The report of a link between childhood leukemia and ELF magnetic fields fueled discussion for more than a decade, ushering together two very different areas, electrical engineering and biology.

The arguments oppose the officers of industry and their followers to health environmentalists. Given that both should have access to the same scientific literature, the opposing positions rely on the selection of different experts, subsets of the literature, and on their interpretation.

This article attempts to explain how different views on the health effects of technological electromagnetic radiation solidified over time, some believing they are negligible, while others propose they are substantial.

We skirt around classically reported events such as the development of the ANSI (21), IEEE (22) and ICNIRP (23) recommendations as well as the Bioinitiative report (24), the ORSAA (25) database, Henry Lai’s literature compilation (26), and the NTP (27) and Ramazzini (28) experiments to discuss other incidents that may seem minor but shed some light on the more human aspects of the formation of opinions about EMR health impacts.

To resolve the complex problem of EMR health impacts, several scientific meetings were held worldwide, seemingly to advance science and develop opinions.

We choose to report events where, over the years, the author was *physically* present; such direct experiences easily solidify into valuable memories. We think that assembling such recollections can be used to explain the development of diverging opinions on EMR health impacts.

## Adair 1991

I attended a presentation by Robert Adair at a Bioelectromagnetics meeting where he reported that the health effects of ELF were incompatible with physics. Adair was a nuclear physicist interested in elementary particles who became popular through his study of the physics of baseball. In his retirement, Adair was involved in the health effects of ELF magnetic fields, as his wife Eleanor Adair presided over the IEEE Committee on Man and Radiation and the IEEE Standards Coordinating Committee 28 (Sub Committee 4) on RF health effects.

Adair’s presentation was based on his 1991 publication titled “Constraints on biological effects of weak extremely low-frequency electromagnetic fields” (2). Adair’s conclusion was that “any biological effects of weak ELF fields on the cellular level must be found outside the scope of conventional physics.” It seems certain that Adair included quantum mechanics in “conventional physics,” since he labeled Lednev’s proposed mechanism for biological magnetic field action (3) “not just bad science…crackpot quantum mechanics.” Adair seemed convinced enough of his position to challenge Lednev, a member of the USSR Academy of Sciences from the Institute of Biological Physics in Puschino [(4) J/F]. However, in April 1991, physicists in the EPA’s Science Advisory Board meeting in San Antonio, presided by Charles Susskind, agreed to delete references to Adair’s article in their recommendations.

Despite the 1991 controversy over Adair’s conclusion that ELF biological effects were a physical impossibility, Adair’s ideas were very well received by the attendees. Bioelectromagnetics meeting participants were industry representatives mixed with academics aware that research funding from industry was sure to enhance their university profiles. Consequently, no one dared to challenge Adair’s industry-friendly comments, partly because the details of a paper are not commonly dissected in a podium presentation.

Reading over the 1991 paper later, I noticed many risky affirmations, but the argument most prominent in Adair’s mind, and the one retained in his abstract, was the following (p. 1043).

From the equation E = B_Earth_ v, according to which a charge in the body of a walking person moving at speed “v” and in the Earth’s magnetic field “B_Earth_” creates an electric field “E,” Adair notes that this field is equal to the “maximum field generated by a 4 μT AC 60-Hz magnetic field” in the body. This implies that being exposed to ELF magnetic fields cannot be more dangerous than taking a walk.

What is not clearly noted in the article or in the abstract of the paper is that since the speed of walking “v” is more or less constant, as is the Earth’s field “B_Earth_,” the induced field E is mostly a DC field, unless one walks at 60 Hz.

Why did Adair, a trained physicist, momentarily lose sight of the distinction between DC and AC fields? One could argue that this is an unforgiveable oversight in the context of such a discussion. Could the electrical engineers in his audience, their discipline rooted in Faraday’s Law (∂B/∂t), gloss over this blunder and retain in their minds the message that ELF fields are inoffensive, as implied by Adair’s reasoning?

Although EPA physicists challenged Adair’s conclusions even as they were published, Adair presented his arguments firmly at a conference. His presentation could be quite influential to an audience already leaning toward the conclusion that ELF biological effects are improbable.

## Armstrong 1994

In 1991, I described an electromagnetic dosimeter (5) used in the Électricité de France-Québec Hydro and Ontario Hydro epidemiological study on cancer in utility workers (6). In 1994, I co-authored the Benedict Armstrong et al. article “Association between exposure to pulsed electromagnetic fields and cancer in electric utility workers in Quebec, Canada, and France” (7).

The dosimeter measured power frequency electric and magnetic fields as well as Pulsed ElectroMagnetic Fields or High-Frequency Transients (PEMF-HFTs). I included the PEMF-HFT detection capability in the dosimeter to widen in frequency the scope of fields compiled in the epidemiological study, initially limited to 60 and 50 Hz. I could justify the added detection capability as a means of monitoring spurious electrical discharges in power networks (corona or flashover faults), and this did not meet with any resistance, as engineers are routinely favorable to gathering more data.

A dramatic turn was taken when Armstrong, an applied biomedical statistician currently working at the Department of Social and Environmental Health Research, London School of Hygiene and Tropical Medicine, reported that PEMF-HFT exposures were associated with lung cancer in utility workers: “There was an association of PEMF-HFTs with lung cancer that was strong by any standards.” (7).

After this disclosure, the university epidemiological team requested an additional funding of 50,000$ to the original 3,000,000$ from the utility to investigate the PEMF-HFT cancer question, but this request was denied. Further, it was required that all the dosimeters and the data gathered be returned to the utility and never used again [(8) Nov/Dec].

Whatever one may think about the PEMF-HFT cancer connection, the events illustrate a confidence gap between the utility and the university epidemiologists. The utility was likely so convinced that the PEMF-HFT cancer connection was invalid that it felt justified to deny the project extension and extinguish any further analysis. Admittedly, *post hoc* revelations, statistical analyses developed after the data are seen, are viewed somewhat skeptically in epidemiology. Perhaps the extra funding was perceived as a desire to extend a contract.

Following Armstrong’s publication, I was asked by the university team to present the cancer findings to IEEE’s Standards Coordinating Committee 28 (Sub Committee 4) chaired by Eleanor Adair. I felt intimidated during that presentation, as I knew of Eleanor Adair’s unwavering skepticism over EMR health effects and fully realized that this kind of news was not what IEEE relished. However, I was somewhat relieved when it was decided that SC4 would form a group to investigate the questions raised by Armstrong’s report. Within hours, I found myself in a room with twenty or so people and a call was made for volunteers to investigate this interesting new PEMF-HFT cancer association.

As the call for three volunteers was made, I raised my hand, and seeing only one other hand, I could hope that I would be part of the action since I had more inside information on the dosimeter than anyone and had been part of the epidemiological team discovering the PEMF-HFT-cancer association.

Despite my name being duly recorded, I would not be involved in further work on PEMF-HFTs within IEEE. And as it turns out, neither Armstrong nor I were asked to participate in the follow-up work of any kind, either with IEEE or with the utility. It is not unheard of for tightly knit groups such as IEEE sub-committees to prefer working on critical problems with individuals that they trust will have the same viewpoint rather than with scientists with less predictable perspectives, a form of confirmation bias. Since the university could not work of PEMF-HFTs using its large original database, only modest work was done (9) on PEMF-HFTs in the following years using Positron dosimeters purchased by the National Institute of Occupational Health in Denmark to assess their occurrence among different populations.

At the utility level, further investigation determined that there was uncertainty as to whether the dosimeter detected PEMF-HFTs or spurious emissions from the walkie-talkies used by power utility personnel (10). This placed the observation of increased cancer rates associated with PEMF-HFTs between two chairs, allowing both the utilities and the telecommunications industry to distance themselves from the problem.

In 1995, EPRI handed a contract to EM Factor and Enertech consultants to develop a more sophisticated PEMF-HFT dosimeter. However, the project encountered practical difficulties in producing a compact instrument that could be used for epidemiological-style studies [(11) Sep/Oct]. It may be that the industry was reluctant to push the agenda on transients because it was known that they could rise above the background thermal noise, which has been a favourite justification for the high protection limits promoted by the industry. “The objection that environmental fields are too weak with respect to thermal noise need not apply to transients,” as stated by Dr. Antonio Sastre, a consultant sponsored by EPRI based in Suffern, NY [(12), p. 5, (30)].

This dead end is remindful of the National Toxicology Program’s conundrum on the issue of cell phone radiofrequency (RF) radiation health effects. Apparently, technical challenges have impaired a follow-up to the NTP [NTP] study that suggested a link between cell phone radiation and cancer (13).

Continuing the research for both PEMF-HFTs and cell phones may be technically challenging, but the potential cost to public health may be far-reaching as, unlike other potential carcinogens, avoidance is not an option. However, the more likely motive is that pushing investigations further into new terrains could produce uncertain results, as is typical in authentic research. Outcomes altering safety perspectives in important industries may not be managerially attractive. And can corporations or government agency managers be blamed for not investing their hard-earned dollars into research that may trigger upheavals?

Around 1994, many researchers, including Robert Becker, used PEMFs to improve tissue regeneration (14). In the dosimeter article, I described the high-frequency data channel as “High Frequency Transients” (HFT) out of deference to the scientific community involved in tissue regeneration. I intended to avoid casting a shadow on the therapeutic applications labeled “PEMF,” which included bone non-union of long bone fractures (15) and other regenerative work. However, in his article, Armstrong did not go for the neologism and substituted PEMF (which was much better known) for HFT. Not unexpectedly, Becker wrote a letter to me expressing his displeasure with the implication that PEMFs could somewhat be linked with cancer.

While PEMFs are still used therapeutically today, the term “HFT” was forgotten, while the impulsive signals of digital telecommunications have been consistently scrutinized for carcinogenicity (16).

This dichotomy of overlapping morbidity and therapy is difficult to resolve. Still, it is clearly linked to the biological context in which fields are applied, and to the intensity, duration and impulsive parameters of the exposures. In RF parlance, this means that modulations and crest factors are significant in determining biological outcomes and that biology reacts according to the time course of exposures.

## Lai 1997

I attended a conference where Lai and Singh presented the results of their article “Acute Exposure to a 60 Hz magnetic Field Increases DNA Strand Breaks in Rat Brain Cells” (17).

Working at the time for the research arm of an electrical utility company, I realized that I could be witnessing a major advance in biophysics. According to the electrical utility views, DNA strand breaks were impossible, because the radiation is “non-ionizing.” Lai gave the last talk in the session, and after he finished, I rushed to the podium for more details, expecting a fight for his attention with other scientists eager to know more. But to my surprise, the room quickly vacated, leaving Lai and myself alone in the room.

What had happened, as reported in Microwave News [(8) XIV No 6, (29) XVII, No 1], is that Lai had already been “war-gamed” by Motorola for his earlier results linking DNA damage with RF radiation. Lai had been ostracized by an industry that called for Lai to be fired from his university position (18).

Motorola’s corporate communications department issued the following statement in relation to Lai’s work:

“While this work raises some interesting questions about possible biological effects, it is our understanding that there are too many uncertainties—related to the methodology employed, the findings that have been reported and the science that underlies them—to draw any conclusions about its significance at this time. Without additional work in this field, there is absolutely no basis to determine whether the researchers found what they report finding—or that the results have anything at all to do with DNA damage or health risks, especially at the frequencies and power levels of wireless communication devices.”

Public relations specialists often call for “additional work in this field” to spin unfavorable scientific results. Replication in biology is nowhere comparable to what it is in the physical sciences, primarily because biological models are alive and constantly changing.

Is the statement above a legitimate parry by industry to Lai’s results? This depends on whether the statement is issued by a lawyer or by an industry scientist.

In the ears of biological and many other scientists, the statement above together with the persecution of Lai reported in the University of Washington Magazine (18) seem a highly hostile response to the publication of a scientific paper.

However, within an industry environment, it is easy to emphasize the uncertainties of bio-science results. There is inevitably a certain impatience with a body of possibly aberrant scientific investigations that challenge current industry safety practices in a very deep way.

My point is that while Lai was busy defending himself from various institutional challenges, these comments solidified the mistrust that the engineering and business communities harbored toward the biological sciences.

Engineering can achieve wonders if supported with proper development resources. Those resources are often in turn determined by investments and very sensitive to regulatory uncertainties that can wipeout profits, as happened in the tobacco and asbestos industries.

Contrast this with the reputation of academia, home of open-ended quests for knowledge. Industry feels its money is hard-earned in the context of heavy competition and is denatured when given as handouts for health research.

## Phillips 2009

I attended a conference where Dr. Phillips presented his paper “Electromagnetic fields and DNA damage” (19). Because of the co-authorship with Lai and Singh, the comet assay, which had been used previously to show damage by ELF and RF radiations would obviously be central to that paper.

Before the conference, I mentioned the paper and the *comet test* to my superior at the power utility where I worked. His answer, obviously relayed from the industry grapevine, was short: “It’s not a good test”.

I withdrew carefully, knowing that my industry boss had no tangible knowledge of biology and that his negative comment on the *comet test* came from his utility contacts. In those years, I had not yet acquired substantial experience in the biology laboratory and could not challenge his view.

Following Phillips’ conference, I arranged to share an elevator ride with him and inquired: “I hear the comet test is not very good”. Phillips instantly quipped back, displaying a scandalized stare: “It’s a very good test”. We both stood there, somewhat embarrassed but knowing full well that in a few seconds the elevator doors would open, ending our misery. This is the shortest scientific conversation I have ever had.

As it turns out, the comet test is very good, an assay of choice worldwide (31). Although more delicate to implement than the micronucleus erythrocyte test, it is much more versatile. Was the reluctance to accept Lai’s results connected with doubts about Singh’s competence or skepticism based on the reliability of general biophysics?

Is this exchange symbolic of the flimsy dialogue between industry and biology? Phillips seemed to think so, as at the end of his article, he had commented, citing Francis Crick, “…scientists who hold theoretically opposed positions may engage in fruitful debate to enhance understanding of underlying principles and advance science in general…there are external factors involving economics and politics that keep this from happening.”

The “external factors involving economics and politics” that keep productive discussion from happening materialize as diverging perceptions of similar events. The diverging perceptions occur because different educational cultures supply different bases from which to form scientific opinions, for example, Maxwell’s equations vs. the theory of evolution.

But Phillips points out that the conflict amounts to a dispute over society’s limited resources, between engineering and biology. It also involves strategic manoeuvring among individuals and between areas of technology to fuel their development at the expense of other areas. And because of these battles, human health can suffer. Even with the best communications skills, interdisciplinary gaps remain. Unless miracles occur, contacts between distally positioned scientific disciplines often remain primitive.

## Epilogue

The four examples above illustrate different aspects of the divergence of opinions. How are the basic terms of reference in the debate interpreted…are AC and DC fields in some ways equivalent (Adair)? Does the Specific Absorption Rate, a heat variable, provide more clarity than electric and magnetic fields? Should the engineering view of the problem smother its biological aspects? Can humans truly be simulated as a salt and sugar solution, as is done in SAR measurements? What of the issues of not pursuing challenging leads (Armstrong), pressuring the messengers of unwelcome observations (Lai) and undermining or underrating the techniques of biology (Phillips)?

In the recounts above, a leitmotiv is a divergent interpretation of the same facts according to personal education and employment. But control of the collective agenda in the public sphere is equally important, participation at scientific meetings by one group versus another can be strongly biased.

The issue of EMR health impacts was viewed as critical to industry interests at certain times, while for the bio-medical community it may have appeared less urgent.

So, was the industry justified in taking control of the debate and limiting it to heat, given that they had acute interests in the outcomes?

Was industry justified in publicizing its thermalist agenda through the IEEE with so many governments worldwide?

Was industry justified in controlling the evolution of EMR research, using its expertise in electromagnetism to dominate the area, while the most critical elements resided in biology and medicine?

The focus on ionization and thermal limits guaranteed peaceful outcomes for industry. The success of microwave ovens apparently crystallized a view of the human body as a dipole rather than a conductor, but this view may not be everlasting (20).

## Data Availability

The original contributions presented in the study are included in the article/supplementary material, further inquiries can be directed to the corresponding author.
